# Robot-assisted cervical screw placement for atlantoaxial fixation

**DOI:** 10.3171/2025.4.FOCVID2510

**Published:** 2025-07-01

**Authors:** Meghana Bhimreddy, A. Daniel Davidar, Arjun K. Menta, Tej D. Azad, Nicholas Theodore, Daniel Lubelski

**Affiliations:** Department of Neurosurgery, Johns Hopkins University School of Medicine, Baltimore, Maryland

**Keywords:** C1–2 fusion, robot-assisted, ExcelsiusGPS, cervical screw, atlantoaxial fixation

## Abstract

Robotic navigation has primarily been used for instrumentation in the thoracic and lumbar spine. The cervical spine, on the other hand, is a less common site for robot-assisted screw placement. Furthermore, the anatomical complexity and presence of adjacent vascular structures in this region necessitates high placement accuracy. This operative video illustrates the placement of C1 lateral mass and C2 pars screws for a posterior C1–2 fusion using the ExcelsiusGPS surgical robot in a patient with spinal cord compression secondary to a retro-odontoid pannus.

The video can be found here: https://stream.cadmore.media/r10.3171/2025.4.FOCVID2510

## Transcript

This presentation will demonstrate cervical screw placement for atlantoaxial fixation with the ExcelsiusGPS Surgical Robotic Guidance System.

### 0:31 Clinical Presentation.

Our patient is an 81-year-old male who presented with severe neck pain. His pain began approximately 2 months ago after he exerted pressure while performing manual labor. Since then, his symptoms have worsened and he has severe pain with rotation of his neck. Currently, he uses a neck brace and cane for support. On physical exam, the patient had mild weakness of the right upper extremity, particularly during right shoulder abduction and right elbow flexion.

### 1:03 Preoperative Imaging—MRI.

T1 and T2 MRI of the cervical spine revealed a retro-odontoid pannus with a cystic component. This mass posteriorly displaced the tectorial membrane and caused mass effect at the cervicomedullary junction. There was also mild cord caliber loss and subtle increased signal, suggesting myelomalacia. We also saw multilevel advanced degenerative disc changes in the cervical spine, with moderate to high-grade neural foraminal narrowing bilaterally from C2 to T1.

### 1:37 Surgical Plan.

The surgical plan was for a posterior C1–2 fixation and C1 laminectomy using the ExcelsiusGPS for cervical screw placement.

The goals of this procedure were to decompress the spinal cord and nerve roots to arrest the patient’s symptoms and prevent further injury. Stabilizing the degenerated segments of the cervical spine will also allow the pannus to resolve on its own.

### 2:04 Mayfield Pinning.

After the patient was brought into the OR, all bony prominences were padded, and the Mayfield cervical pins were applied.

### 2:12 Prone Positioning.

The patient was then placed in a prone position on the Jackson table, and the Mayfield frame was secured in the cervical management system with care not to hyperextend the neck. Arms were secured to the side using tape while ensuring that no pressure was placed on the brachial plexus. The head of the bed was also elevated slightly.

### 2:31 Intraoperative Neuromonitoring.

Intraoperative neuromonitoring was used during this procedure.

### 2:37 Marking the Surgical Site.

The occipital protuberance and C2 were identified with palpation, and the surgical site was marked, prepped, and draped.

### 2:47 Vertical Incision.

A vertical incision was made from the base of the occiput to C2.

### 2:52 Subperiosteal Dissection.

Subperiosteal dissection was performed with care not to disrupt the extensor muscles attached to the C2 lamina. Bilateral C2 nerve roots were ligated to optimize exposure for screw placement.

### 3:05 Globus Spinous Process Clamp.

The Globus spinous process clamp was placed on C2 and angled toward the head.

### 3:11 Patient Reference and Registration Arrays.

The patient reference and registration arrays were then placed.

### 3:17 O-Arm.

The O-arm was brought in for intraoperative CT. This data was then transferred to the robot. The accuracy of registration was determined by using bony landmarks, and, once confirmed, the registration array overlying the surgical wound was removed.

### 3:34 Planning Screw Trajectories.

Bilateral C1 lateral mass screws and bilateral C2 pars screws were then planned and navigated, and the robot was brought into the field.

### 3:43 Confirming Registration with Navigated Instrument.

A navigated instrument was used again to confirm the accuracy of registration.

### 3:49 Robotic Arm Moving Into Position.

In this operative video, we will show the placement of the right C1 lateral mass screw and right C2 pars screw, starting with the former. First, the robotic end effector arm was brought into place.

### 4:02 Drilling of the C1 Lateral Mass Pilot Hole.

Next, the pilot hole was drilled.

### 4:07 Cannulation of the C1 Lateral Mass Pilot Hole.

The pilot hole was then cannulated.

### 4:13 Placement of the C1 Lateral Mass Screw.

This hole was palpated with a feeler to ensure no breach had occurred. Then, the C1 lateral mass screw was placed.

### 4:23 Robotic Arm Moving Into Position.

We followed the same procedure for the right C2 pars screw. The robotic arm was moved into position.

### 4:31 Drilling of the C2 Pars Pilot Hole.

The C2 pars pilot hole was then drilled.

### 4:36 Cannulation of the C2 Pars Pilot Hole.

The pilot hole was then cannulated.

### 4:42 Placement of the C2 Pars Screw.

After palpating the hole with a feeler to ensure no breach occurred, the C2 pars screw was placed.

### 4:56 Image.

The left C1 lateral mass and C2 pars screws were placed in a similar fashion. One important note is that C2 screw placement can experience lateral deflection from muscle pushing the drill, especially with a small incision. In our case, the patient habitus was favorable and allowed for adequate retraction with a modest incision. Furthermore, navigation guidance allowed for planning of screw trajectories that were straight in and not medially directed, which further minimized the need for a medialized trajectory.

### 5:29 Rod Cutting.

After all screws were placed, 3.5-mm rods were cut.

### 5:35 Rod Placement.

They were placed to connect the C1 to C2 screws on each side.

### 5:42 Screw Cap Placement.

The screws caps were placed and tightened.

### 5:45 C1–2 Laminectomy.

A C1–2 laminectomy was performed and ultrasound was used to confirm there was excellent decompression. The C1–2 articular surface was decorticated with a high-speed drill. Local bone and synthetic allograft were placed posterolaterally to promote fusion.

### 6:03 Image.

An intraoperative x-ray was obtained to confirm placement of our instrumentation.

### 6:09 Drain Placement and Closure.

Vancomycin powder was placed in the wound and a Hemovac drain was inserted in the deep layer. The muscle, fascia, subdermal, deep dermal, and skin layers were then closed. Skin glue was applied to the site after closure and allowed to dry.

### 6:25 CT Images.

We also obtained a postoperative CT scan, which showed excellent placement of the cervical screws.

### 6:33 Postoperative Course.

After the procedure, the patient was admitted to the neurosurgery floor for postoperative care. He was discharged home on postop day 1 with no complications. At his 2-week follow-up clinic visit, the patient reported that he was able to carry out all of his regular home activities again. He was off pain meds and reported an increased subjective sense of strength and decreased paresthesias. He showed no weakness on physical exam.

### 7:00 Alternatives.

Currently, the most commonly used approach for this procedure is freehand screw placement using direct visualization and fluoroscopy. This has an accuracy rate of around 80%. However, this technique requires technical mastery and a detailed understanding of the anatomy surrounding the occipitocervical junction. Our institution favors the freehand approach for cervical screw placement, though robotic assistance is more common if patients have abnormal anatomy or require placement of transarticular screws. Other alternatives include navigation and augmented reality–based techniques.

### 7:41 Benefits of Robotic Assistance.

Robotic assistance for cervical screw placement offers a variety of benefits over traditional methods. For example, the fixed robotic end effector arm allows the surgeon to follow the same trajectory for every step of the screw placement process without having to reorient in between. It also reduces the amount of deviation or deflection during the use of power tools, which can be helpful when working in narrow corridors such as those in the cervical spine. Current literature indicates that robotic screw placement in the cervical spine leads to higher perfect screw accuracy, defined as GRS A, than the freehand approach. It was also found to significantly reduce complication rates, intraoperative blood loss, length of hospitalization, and radiation dose. Recently, studies have also been published demonstrating the high accuracy of robot-assisted cervical transarticular and transodontoid screw placement.

### 8:46 Risks of Robotic Assistance.

Currently, robotic assistance is primarily used for screw placement in the thoracolumbar and sacral regions. Therefore, adopting this approach for cervical screw placement may lead to increased operative time initially. However, the learning curve associated with robot-assisted screw placement is well documented, with multiple studies indicating notable reductions in per-screw time and fluoroscopy time with each case. Another consideration is the initial cost associated with acquiring a robotic system. Finally, care must be taken to prevent reference array movement during robotic procedures. If movement occurs, the robot will interpret this as patient movement, leading to improper screw placement. At our institution, we find that placing screws at the level where the array is attached may cause some movement. Therefore, we perform screw placement at this level last. In this case, our reference array was attached to C2. Therefore, we placed the C2 pars screws after the C1 lateral mass screws.

### 9:54 References. ^1–10^

## Disclosures

Dr. Theodore reported personal fees from Globus Medical outside the submitted work. Dr. Lubelski reported personal fees from Carbofix, Icotec, Dilon Technologies, Mindset Medical, and Globus outside the submitted work.

## Author Contributions

Primary surgeon: Theodore, Lubelski. Assistant surgeon: Azad. Editing and drafting the video and abstract: Bhimreddy, Davidar, Menta, Theodore, Lubelski. Critically revising the work: all authors. Reviewed submitted version of the work: Bhimreddy, Davidar, Menta, Lubelski. Approved the final version of the work on behalf of all authors: Bhimreddy. Supervision: Azad, Theodore.

## Supplemental Information

### Patient Informed Consent

The necessary patient informed consent was obtained in this study.
